# Antibacterial effects of Traditional Chinese Medicine monomers against *Streptococcus pneumoniae* via inhibiting pneumococcal histidine kinase (VicK)

**DOI:** 10.3389/fmicb.2015.00479

**Published:** 2015-05-20

**Authors:** Shuai Zhang, Jianmin Wang, Wenchun Xu, Yusi Liu, Wei Wang, Kaifeng Wu, Zhe Wang, Xuemei Zhang

**Affiliations:** Key Laboratory of Diagnostic Medicine Designated by the Ministry of Education, Department of Laboratory Medicine, Chongqing Medical UniversityChongqing, China

**Keywords:** *Streptococcus pneumoniae*, Traditional Chinese Medicine monomer, antimicrobial, histidine kinase, VicK protein

## Abstract

Two-component systems (TCSs) have the potential to be an effective target of the antimicrobials, and thus received much attention in recent years. VicK/VicR is one of TCSs in *Streptococcus pneumoniae* (*S. pneumoniae*), which is essential for pneumococcal survival. We have previously obtained several Traditional Chinese Medicine monomers using a computer-based screening. In this study, either alone or in combination with penicillin, their antimicrobial activities were evaluated based on *in vivo* and *in vitro* assays. The results showed that the MICs of 5′-(Methylthio)-5′-deoxyadenosine, octanal 2, 4-dinitrophenylhydrazone, deoxyshikonin, kavahin, and dodecyl gallate against *S. pneumoniae* were 37.1, 38.5, 17, 68.5, and 21 μg/mL, respectively. Time-killing assays showed that these compounds elicited bactericidal effects against *S. pneumoniae* D39 strain, which led to a 6-log reduction in CFU after exposure to compounds at four times of the MIC for 24 h. The five compounds inhibited the growth of *Streptococcus pyogenes, Streptococcus mitis, Streptococcus mutans* or *Streptococcus pseudopneumoniae*, meanwhile, deoxyshikonin and dodecyl gallate displayed strong inhibitory activities against *Staphylococcus aureus*. These compounds showed no obvious cytotoxicity effects on Vero cells. Survival time of the mice infected by *S. pneumoniae* strains was prolonged by the treatment with the compounds. Importantly, all of the five compounds exerted antimicrobial effects against multidrug-resistant clinical strains of *S. pneumoniae*. Moreover, even at sub-MIC concentration, they inhibited cell division and biofilm formation. The five compounds all have enhancement effect on penicillin. Deoxyshikonin and dodecyl gallate showed significantly synergic antimicrobial activity with penicillin *in vivo* and *in vitro*, and effectively reduced nasopharyngeal and lung colonization caused by different penicillin-resistant pneumococcal serotypes. In addition, the two compounds also showed synergic antimicrobial activity with erythromycin and tetracycline. Taken together, our results suggest that these novel VicK inhibitors may be promising compounds against the pneumococcus, including penicillin-resistant strains.

## Introduction

Invasive pneumococcal infections, such as pneumonia, sepsis, and meningitis, commonly have high morbidity and mortality worldwide (Bandettini and Melioli, 2012). In recent years, the emergence of multidrug-resistant and vancomycin-resistant strains has become increasingly frequent because of the antibiotics abuse in hospitals (Hanna-Wakim et al., 2012). Therefore, the development of novel antimicrobials against pneumococcal infections is indispensable.

Two-component systems (TCS) are deserved to be considered as effective drug targets, because they are required for the regulation of bacterial virulence and growth (Barrett and Hoch, 1998; Fabret and Hoch, 1998) and TCSs have not been found in mammals (Worthington et al., 2013). TCSs are composed of a sensor kinase (histidine kinase, HK) and a response regulator. Compared with drugs that target the sensory domains of HKs, those that target the conserved catalytic domains of HKs which could coordinately inhibit multiple TCSs are expected to reduce the virulence of pathogenic microorganisms more efficiently (Gotoh et al., 2010). Yamamoto and Watanabe (Yamamoto et al., 2001; Watanabe et al., 2003) have verified that HK inhibitors, such as synthetic imidazole, zerumbone derivatives, and aranorosinol B, were effective antibacterial agents against *Bacillus subtilis*.

VicK/VicR is conserved and specific to low G+C Gram-positive bacteria. It has been reported that VicK/VicR is essential for the survival of pathogens, such as *Streptococcus pneumoniae* (*S. pneumoniae*) (Wayne et al., 2012) and *Staphylococcus epidermidis* (*S. epidermidis*) (Qin et al., 2007). Qin et al. has previously reported that seven compounds showed antibacterial activities against *S. epidermidis* strains by targeting VicK *in vitro* (Qin et al., 2006). We have also previously obtained six synthetic compounds with effective antibacterial activity that targeted the ATPase domain of VicK in *S. pneumoniae* (Li et al., 2009).

Traditional Chinese Medicine (TCM) may be an important source of new drugs. We have previously used a structure-based virtual screening method to screen the natural TCM monomers with the ability to interact with VicK protein by targeting the VicK HATPase_c domain, and the screening procedure was essentially performed according to our previous established method except the change in drug database (Li et al., 2009). A total of 96 natural TCM monomers were identified as potential inhibitors of the VicK protein. In the present study, five compounds were identified as VicK inhibitors. They were 5′-(Methylthio)-5′-deoxyadenosine, octanal 2, 4-dinitrophenylhydrazone, deoxyshikonin, kavahin, and dodecyl gallate. 5′-(Methylthio)-5′-deoxyadenosine is extracted from *Saccharomyce scerevisiae*. Octanal 2, 4-dinitrophenylhydrazone is the compound from extracts of a variety of plants such as honeysuckle. Deoxyshikonin, an extract from the roots of Maharanga, was identified to exhibit anticancer activity (Rajasekar et al., 2012). And kavahin, extracted from the root of kava, was reported as an analgesic agent (Kormann et al., 2012). To the best of our knowledge, there is no related report about the antibacterial activity of these 4 compounds. Only dodecyl gallate has been reported as an antibacterial agent against methicillin-resistant *Staphylococcus aureus* (MRSA) (Kubo et al., 2003), but it remains unknown whether dodecyl gallate has antibacterial effect against other bacteria.

In this study, the five compounds were primarily tested for their efficacies against pneumococcal strains including penicillin (PEN)-resistant *S. pneumoniae* (PRSP), and their MIC concentrations were determined. To investigate whether they have a broad antibacterial effect, the five compounds were tested for their efficacies against MRSA and other streptococci. Their efficacies against pneumococcal infections were also evaluated in mouse sepsis models and local infection models. In addition, the mechanism involved in the antibacterial effect was explored.

## Materials and methods

### Bacterial strains and plasmids

The strains and plasmids used in this study are listed in Table 1. *Escherichia coli* (*E. coli*) and *Staphylococcus aureus* (*S. aureus*) strains were grown in LB medium. *S. pneumoniae* strains were grown in C+Y medium at 37°C in 5% CO_2_. Other streptococci strains were grown in THB medium.

**Table 1:** **Bacterial strains and plasmids used in this study**.

**Strain or plasmid**	**Relevant characteristics**	**Source or reference**
**STRAINS**		
***E. coli***		
BL21 (DE3)	F ompT hsdSB (rBmB) gal dcm (DE3)	Novagen
***S. pneumoniae***		
NCTC7466 (D39)	*S. pneumoniae* reference strain	National Collection of Type Cultures (London, UK)
CMCC31109	Serotype 1	National Center for Medical Culture Collections (CMCC Beijing, China)
CMCC31203	Serotype 3	
CMCC31207	Serotype 6B	
CMCC31446	Serotype 4	
CMCC31614	Serotype 14	
CMCC31689	Serotype 19A	
CMCC31693	Serotype 19F	
Clinical isolates (34)		
*Streptococcus pyogenes*	Clinical isolates	Children's hospital, Chongqing, China
*Streptococcus mitis*	Clinical isolates	
*Streptococcus mutans*	Clinical isolates	
*Streptococcus pseudopneumoniae*	Clinical isolates	
***S. aureus***		
ATCC29213	*S. aureus* reference strain	American type culture collection (Maryland, USA)
Clinical isolates (3)		Children's hospital, Chongqing, China
**PLASMIDS**		
pEVP3		Lee and Morrison, 1999
pEVP3-*ftsW*		Yan et al., 2013

### Traditional chinese medicine monomer

**Figure 1:**
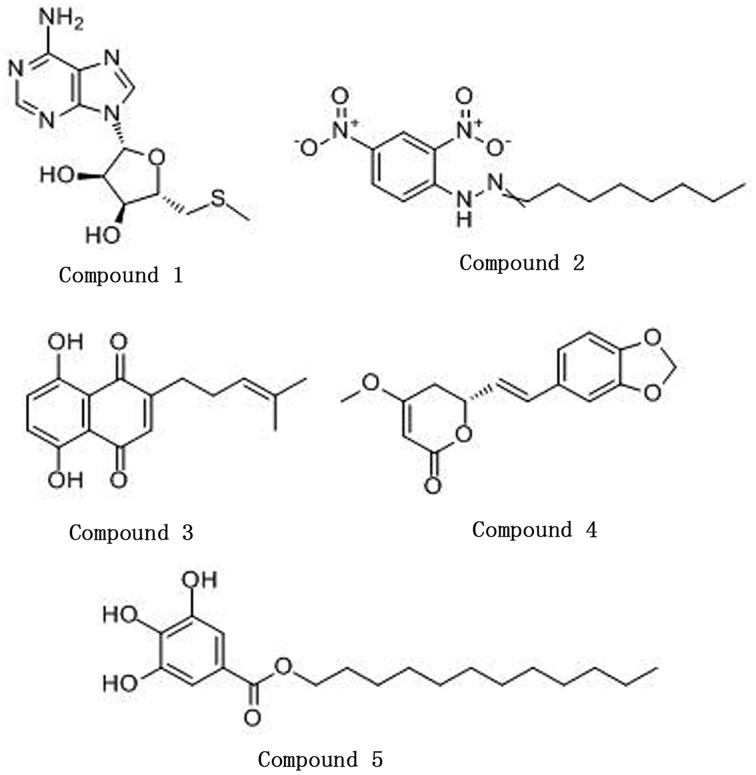
**The structures of the five compounds**. Compound 1, 5′-(Methylthio)-5′-deoxyadenosine; Compound 2, Octanal 2, 4-dinitrophenylhydrazone; Compound 3, Deoxyshikonin; Compound 4, Kavahin; Compound 5, Dodecyl gallate.

5′-(Methylthio)-5′-deoxyadenosine (CAS: 2457-80-9), octanal 2, 4-dinitrophenylhydrazone (CAS: 1726-77-8), and dodecyl gallate (CAS: 1166-52-5) were purchased from Sigma. Deoxyshikonin (CAS: 43043-74-9) and kavahin (CAS: 495-85-2) were purchased from J&K Scientific and Tautobiotech, respectively. Stock solutions of the monomers were prepared in dimethyl sulfoxide (DMSO). The structural formulas of the monomers are listed in Figure 1.

### Cytotoxicity assay

The cytotoxicity of the drugs to Vero cells (African green monkey kidney cells) was detected as previously described (Huang et al., 2012) with minor modifications. In 96-well cell culture plates, the cells (~10^3^ cells per well) were exposed to the serially diluted drugs for 48 h. Then, relative cell numbers were assayed by co-incubation with 3-(4, 5-dimethylthiazol-2-yl)-2, 5-diphenyltetrazolium bromide for 4 h at 37°C in 5% CO_2_. Purple formazan salts were dissolved with DMSO after incubation. The absorbance of each well was measured at 595 nm and then converted to percentages of the control (cells treated with 1% DMSO). The concentration of the derivatives with 50% cytotoxicity for Vero cells (CC_50_) was calculated using GraphPad Prism 5 (San Diego, CA).

### Inhibition assay for ATPase activity

The inhibitory activities of the Chinese medicine monomers for the ATPase activity of the full-length VicK protein were evaluated using the Kinase-Glo™Luminescent Kinase Assay (Promega, Madison, USA). Briefly, 6 μg VicK protein was pre-incubated with a series of dilution of compounds in the reaction buffer at 37°C for 30 min. 5 μM ATP was added and incubated for another 30 min. Kinase-Glo™Reagent was also added to detect the remaining ATP recorded from luminescence measurements (RLU). In parallel, the VicK protein with no addition of compounds was used as a control, and the mixture with compounds excluding the VicK protein served as another control. The rate of protein phosphorylation (Rp) inhibition by the compounds was calculated from Equation (1).

(1) $$\begin{array}{l} \text{Rp}=1-\text{RLU}(\text{compound}+\text{ATP}+\text{Reagent})\\ \;-\text{RLU}(\text{compound}+\text{ATP}+\text{vick}+\text{Reagent})/\text{RLU}(\text{ATP}\\ \;+\text{Reagent})-\text{RLU}(\text{ATP}+\text{vick}+\text{Reagent})\times 100\% \end{array}$$

The half maximal inhibitory concentration (IC_50_), which is the concentration of inhibition of 50% VicK protein autophosphorylation, was determined by GraphPad Prism 5 (Qin et al., 2006).

### Antimicrobial susceptibility testing

Minimum inhibitory concentration (MIC) assays for the antibacterial activities of the compounds were performed according to the standard agar dilution method of the Clinical and Laboratory Standards Institute of America. MIC testing of drug-resistant *S. pneumoniae*, drug-resistant *S. aureus*, streptococci and antimicrobial combination was also performed using the same method. Briefly, the drugs were serially diluted 2-fold into eight wells with C+Y medium, yielding final concentrations ranging from 2000 to 1.5 μM. Then 10^6^ CFU bacteria were added to each well. Inoculated medium containing 1% DMSO served as a bacterial growth control, whereas uninoculated medium served as a sterility control. The plates were incubated at 37°C for 12–16 h. The lowest concentration that completely inhibits visible growth of the organism as detected by the unaided eye was recorded as the MIC (Giacometti et al., 2000). The experiment was repeated three times.

For time-kill assays, the organisms were cultured at 37°C for 1 h and then treated with the drugs at one-fourth, one, and four times the MIC. Viable cells were counted at 0, 2, 4, 6, 8, and 24 h after the addition of the compounds (Mun et al., 2013).

### Determination of the *in vitro* effects of combinations of antimicrobial agents

The antimicrobial effects of different combinations of two antimicrobial agents were assayed using the checkerboard test (Bajaksouzian et al., 1997). Each experiment was repeated thrice. The *in vitro* interaction between the five drugs with PEN was quantified by the fractional inhibitory concentration (FIC). The FIC index (FICI) was calculated using the following formula:

(2) $$\text{FIC}={\text{FIC}}_{\text{A}}+{\text{FIC}}_{\text{B}}=[\text{A}]{\text{/MIC}}_{\text{A}}\text{+}[\text{B}]{\text{/MIC}}_{\text{B}}$$

where [A] and [B] are the concentrations of drugs A and B, respectively; MIC_A_ and FIC_A_ are the MIC and FIC of drug A for the organism, respectively; and MIC_B_, and FIC_B_ are the MIC and FIC of drug B for the organism, respectively. The FIC index obtained was interpreted as follows: =0.5, synergy; FIC > 2.0, antagonism (Gupta and Kohli, 2003).

### Biofilm-killing assays

Biofilm formation was detected using a semiquantitative plate assay (Moscoso et al., 2006). 10^6^ CFU of D39 was incubated in C+Y medium for 6 h in a 96-well plate at 37°C. Then removed the planktonic cells, fresh C+Y medium containing the serially diluted drugs was added to each well and incubated for another 12 h at 37°C. The wells were washed gently three times with phosphate-buffered saline (PBS) and then stained with 1% (w/v) crystal violet. 200 μL methanol was added to dissolve the precipitate and determine the absorbance of the wells at 590 nm using a spectrophotometer (DTX880, Beckman Coulter, USA). The values of biofilm formation were normalized for absorbance, and the percentages were calculated in relation to control. The experiment was repeated twice.

### Cell division phenotype assays

Phenotype assays were performed as previously described. Briefly, overnight bacterial cultures were used for inoculation (1:1000 dilutions). Then, 150 μL aliquots were added to 96-well microtiter plates containing dilutions of each compound in 50 μL volumes of medium. After incubation for 5 h at 37°C, 10 μL cultures were blotted to glass slides and then stained with leather blue dye solution for microscopic analysis. For Transmission electron microscopy (TEM), bacteria were collected by centrifugation at 4°C for 10 min at 10,000 g. Precipitates were fixed in 2% glutaraldehyde in sodium cacodylate buffer (pH 7.4) for 12 h and then processed by the Electron Microscopy Research Service of Chongqing Medical University. For visualization, cells were imaged with a Hitachi H-7500 transmission electron microscopy.

### β-galactosidase reporter gene assay

*S. pneumoniae* D39 and D39-pEVP3-*fstW* containing FtsW::LacZ fusion were cultured in C+Y medium until OD_600_ = 0.5–0.6. Subsequently, 10^7^ CFU bacteria were collected by centrifugation at 13,800 g for 2 min. The pellet was collected and washed twice with PBS. The pellet was resuspended in 500 μL of 0.1% triton X-100 dissolved in PBS and then incubated for 15 min at 37°C. 50 μL of the reaction product was used for detection following the instructions of β-galactosidase reporter gene assay kit (Beyotime, China). Finally, the protein expressions were detected using a micro plate reader at 450 nm.

### *In vivo* experiments

All animal experiments were approved by the Ethics Committees of Chongqing Medical University. Animals were anesthetized with ethyl ether before any challenges or treatments to minimize their suffering.

Female BALB/c mice (12–15 per group) aged 4–6 weeks were used to evaluate the antimicrobial effects of the compounds *in vivo*. In sepsis models, the mice were intraperitoneally infected with 100 μL *S. pneumoniae* NCTC7466 strain suspension (1 × 10^3^ CFU). An hour later, 100 μL drugs at concentrations of 2 mM (16 × MIC for compound 1, 8 × MIC for compound 4 and 32 × MIC for compound 5) or 4 mM (32 × MIC for compound 2 and 64 × MIC for compound 3) were intraperitoneally injected to the mice. These injections were continued, if the animals did not die, thrice a day for 3 d. Two control groups were injected with 100 μL 1% DMSO (dissolved the drugs, negative control) or PEN (0.42 mg/kg/d, positive control) following the same injection route. The result was expressed as cumulative survival times following 7 d of observation.

In colonization models, the mice were intranasally challenged by 20 μL of clinical drug-resistant strains (pneumococcal serotype 19F or 19A, 1.5 × 10^8^ CFU). An hour later, the mice were intraperitoneally injected with 100 μL drugs (2 × MIC) alone or in combination with PEN (2 × MIC). These injections were continued thrice a day for 3 d. Nasal wash and lung tissues were obtained to analyze the bacteria load at 24, 48, and 72 h after drug infection. Then 100 μL aliquots of the samples diluted were plated on blood agar plates. The colonies were counted after incubation overnight at 37°C with 5% CO_2_.

### Statistical analysis

All experiments were analyzed in three independent assays. Data on the antimicrobial effects of the compounds *in vivo* are shown as means ± SEM of two independent experiments. Unless otherwise stated, statistical analyses of *in vitro* and *in vivo* experiments were carried out using Student's *t*-test (GraphPad Prism 5). *P* < 0.05 was considered to indicate statistical significance.

## Results

### Inhibitory effects of the five compounds on the ATPase activity of vick protein

**Figure 2:**
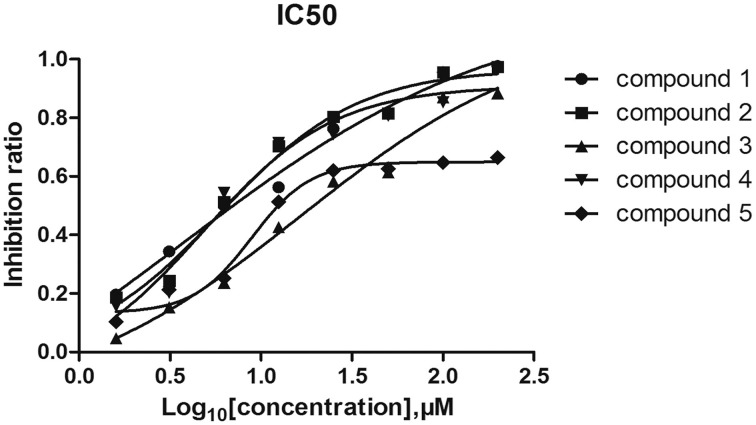
**Inhibitory effects of the compounds on the ATPase activity of VicK**. The inhibitory potencies of the five compounds to VicK were determined at varied concentrations (from 1.6 to 200 μM). Error bars represent the SEM from three independent assays.

6 His-tagged VicK was expressed and purified by Ni^2+^ affinity chromatography. To estimate the inhibitory effects of the five compounds on VicK, purified VicK was incubated with the compounds and its ATPase activity was determined in presence of the compounds (Shuai et al., 2014). The results showed that all five compounds inhibited the ATPase activity of VicK in a dose-dependent manner (Figure 2). The *IC*_50_-values of the five compounds were 3.8 μM (1.12 μg/mL), 5.4 μM (1.65 μg/mL), 15.4 μM (4.2 μg/mL), 4.6 μM (1.25 μg/mL), and 9.1 μM (3.07 μg/mL), respectively. These results demonstrated that each of the five compounds was capable to inhibit the ATPase activity of VicK.

### Antimicrobial activity of the five compounds *In vitro*

The *in vitro* antimicrobial activities of the five compounds and PEN against *S. pneumoniae*, PEN-resistant clinical strains are summarized in Table 2. The five compounds displayed an obvious antimicrobial effect against both PEN-susceptible and PEN-resistant clinical strains. The MIC_50_ values of deoxyshikonin and dodecyl gallate were 17 and 42 μg/mL against PEN-resistant strains, which were 8-fold and 4-fold lower than those of other antimicrobial agents, respectively. These results clearly showed that the five compounds were effective to inhibit bacterial growth, even for strains resistant to PEN.

**Table 2:** *****In vitro*** inhibitory activities of the five compounds against pneumococcal strains**.

**Antimicrobial agent**	**MIC of pneumococcal strains (μg/ml)**								
	**Penicillin susceptible (*n* = 7)**			**Penicillin intermediate (*n* = 12)**			**Penicillin resistant (*n* = 15)**		
	**Range**	**MIC_50_**	**MIC_90_**	**Range**	**MIC_50_**	**MIC_90_**	**Range**	**MIC_50_**	**MIC_90_**
5′-(Methylthio)-5′-deoxyadenosine	74–148	74	148	74–594	148	296	296.8–593.6	296.8	296.8
Octanal, 2-(2, 4-dinitrophenyl) hydrazone	76–152	152	152	38–154	77	154	77–616	308	616
Deoxyshikonin	17–34	17	34	34–196	68	196	8.5–136	17	68
Kavahin	34–272	68.5	136	68–518	137	274	68.5–548	274	548
Dodecylgallate	21–168	42	42	42–168	84	168	10.5–168.8	42	168.8

*n, Indicates the strain numbers*.

As far as we know, VicK is extremely conserved in the low G + C content of gram-positive bacteria, such as *Streptococcus pyogenes* (*S. pyogenes*), *Streptococcus mitis* (*S. mitis*), *Streptococcus mutans* (*S. mutans*), and *Streptococcus pseudopneumoniae* (*S. pseudopneumoniae*). Therefore, the antibacterial activities of the five compounds against other gram-positive strains were assayed in this work. As shown in Table 3, five compounds all exhibited antibacterial effect against *S. pyogenes, S. mitis, S. mutans, S. pseudopneumoniae*, and *S. aureus*. Importantly, deoxyshikonin and dodecyl gallate also showed significantly inhibitory activities against MRSA. These results demonstrated that the five compounds, especially the deoxyshikonin and dodecyl gallate, have broad-spectrum antibacterial effect against gram-positive bacterial infections.

**Table 3:** **Inhibitory activities of five compounds against streptococci and ***S. aureus*** assayed ***in vitro*****.

**Bacteria (No. of isolates)**	**MIC (μg/ml)**				
	**5′-(Methylthio)-5′-deoxyadenosine**	**Octanal, 2-(2, 4-dinitrophenyl) hydrazone**	**Deoxyshikonin**	**Kavahin**	**Dodecylgallate**
*S. pyogenes*	148	308	8.5	274	42
*S. mitis*	148	308	125	274	338
*S. mutans*	594	616	34	274	42
*S. pseudopneumoniae*	148	154	136	34	42
*Methicillin resistant S. aureus (4)*	–^*^	–	34–68	–	84.4–336

*MICs to inhibit the growth of 10^6^CFU/ml bacteria were determined by the 2-fold agar dilution method*.

* *No antibacterial effect when the compounds were tested in the concentrations used in this study*.

**Figure 3:**
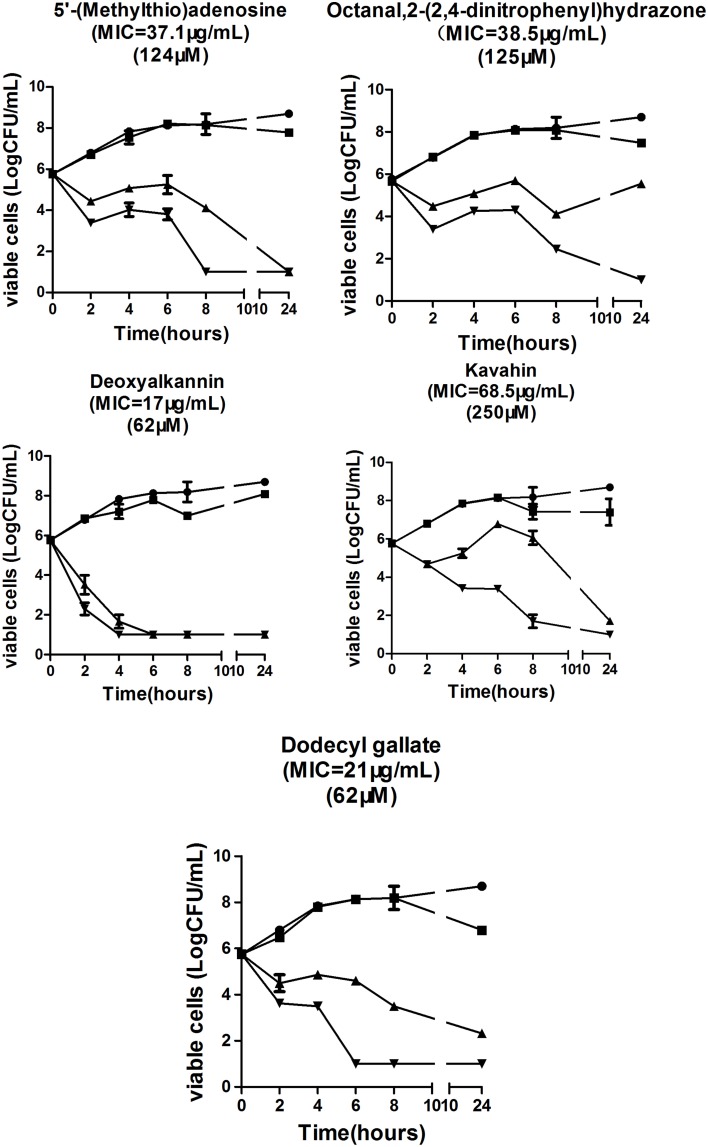
**Bactericidal activities of the five compounds against the strain ***S. pneumoniae*** NCTC7466**.

To further elucidate their antibacterial activities, a time-kill assay was performed to observe the effect of five compounds targeting VicK on bacterial growth. As expected, the five compounds caused a maximum 5-log CFU reduction in *S. pneumoniae* NCTC7466 (Figure 3) at MIC and four times the MIC after 24 h of incubation. These results showed that the five compounds have significant antibacterial activities against *S. pneumoniae in vitro*.

### *In vivo* antimicrobial activity

MTT assay (cc_50_) indicates that the five compounds displayed low toxicity on Vero cells. The therapeutic efficacy of the five compounds against *S. pneumoniae* was determined (Table 4) *in vivo*. Generally, the mice infected with *S. pneumoniae* NCTC7466 (1 × 10^3^ CFU) could not survive more than 30 h without any treatment. Compared with the negative control, although these compounds could not reverse the fatal infection of the concentration used in the present study, they were able to significantly prolong the survival times of the infected mice (*P* < 0.05). Notably, the average survival time of the infected mice was extended to 71 h following the treatment with the kavahin. The results suggested that they may be good compounds in the development of drugs against pneumococcal infection.

**Table 4:** *****In vivo*** inhibitory activities of the five compounds against ***S. pneumoniae*** NCTC7466**.

**Compound**	**Mean survival of mice ± SEM (hours)**	***P*-value^b^**	**Dose (mg/kg)**	**cc_50_** **mM**
Untreated control**^a^**	28.8 ± 1.25			
5′-(Methylthio)-5′-deoxyadenosine	49.5 ± 3.93	0.0062	97.2	>16 (336)
Octanal, 2-(2, 4-dinitrophenyl) hydrazone	42.1 ± 4.1	0.0053	50.4	>4 (<16)
Deoxyshikonin	50.6 ± 4.1	<0.0001	44.5	4.68 (<16)
Kavahin	71.1 ± 7.1	<0.0001	89.7	>8 (193)
Dodecylgallate	60.2 ± 7.3	0.0005	55.3	6.45 (29.5)

a *Infected mice were treated with 1% DMSO (vol/vol) and 0.2% Tween 20 (vol/vol) in water*.

b *Unpaired t-test (vs. untreated control). P < 0.05 was considered significant*.

### Synergic antimicrobial activity of the five compounds and pen *in vitro* and *in vivo*

Given that the five compounds were effective against PEN-resistant clinical strains (Table 2), we determined whether these compounds show synergism with PEN. We investigated the antimicrobial effects of the compounds when combined with PEN using the checkerboard test. Table 5 shows the combined effects of the five compounds and PEN against PEN-resistant *S. pneumoniae* and *S. aureus*. Although only deoxyshikonin and Dodecyl gallate showed significantly synergic antimicrobial activity with PEN against some PEN-resistant *S. pneumoniae* with FICIs ranging from 0.17 to 0.34, all *S. pneumoniae* organisms examined exhibited a 2-fold to 4-fold reduction in MIC values after the treatment with the combination of compound and PEN. Meanwhile, the MICs of deoxyshikonin or dodecyl gallate in combination with PEN against the four strains of MRSA showed a 2-fold reduction.

**Table 5:** *****In vitro*** inhibitory activity of five respective compounds in combination with penicillin against ***S. pneumoniae*** and ***S. aureus*****.

**Strains**	**MIC (μg/ml)**				
	**Agent**	**Alone**	**Compound + penicillin**	**FICI^a^**	**Outcome**
***S. pneumoniae*^b^**					
12092624	Compound1/Penicillin	296.8/3.56	148/0.19	0.55	
	Compound2/Penicillin	154/3.56	77/0.3	0.60	
	Compound3/Penicillin	136/3.56	34/0.19	0.30	Synergy
	Compound4/Penicillin	274/3.56	137/0.19	0.55	
	Compound5/Penicillin	84.4/3.56	42/0.19	0.30	Synergy
736007	Compound1/Penicillin	296.8/7.1	148/2.67	0.87	
	Compound2/Penicillin	154/ 7.1	77/0.6	0.59	
	Compound3/Penicillin	136/7.1	34/0.6	0.34	Synergy
	Compound4/Penicillin	274/7.1	137/0.6	0.59	
	Compound5/Penicillin	337/7.1	84/0.6	0.59	
8364981	Compound1/Penicillin	148/3.56	74/3.1	1.37	
	Compound2/Penicillin	77/3.56	38.5/0.19	0.55	
	Compound3/Penicillin	136/3.56	62.5/0.19	0.17	Synergy
	Compound4/Penicillin	548/3.56	137/0.38	0.26	
	Compound5/Penicillin	168/3.56	84/0.19	0.30	Synergy
652336	Compound1/Penicillin	296.8/4.4	148/0.22	0.55	
	Compound2/Penicillin	154/4.4	77/0.44	0.51	
	Compound3/Penicillin	136/4.4	17/0.22	0.30	Synergy
	Compound4/Penicillin	548/4.4	274/0.44	0.51	
	Compound5/Penicillin	84.4/4.4	42.2/0.44	0.51	
***S. aureus*^c^**					
ATCC29213	Compound3/Penicillin	34/52	17/4.4	0.58	
	Compound5/Penicillin	168/52	84/4.4	0.58	
MRSA8506305	Compound3/Penicillin	34/6682	17/833	0.6	
	Compound5/Penicillin	337/6682	168/333	0.9	
MRSA 8569082	Compound3/Penicillin	68/1667	34/208	0.60	
	Compound5/Penicillin	84.4/166	42.2/208	0.62	
MRSA8548991	Compound3/Penicillin	34/3339	17/416	0.62	
	Compound5/Penicillin	84.4/3339	42/416	0.62	

a *Synergy is defined as FICI < 0.5; Antagonism is defined as FICI > 2*.

b *The clinical strains 12092624, 736007, 8364981, 652336 were Penicillin-resistant S. pneumoniae strains*.

c *The clinical isolates 8506305, 8569082, 8548991 were methicillin-resistant S. aureus strains. Compound1, 5′-(Methylthio)-5′-deoxyadenosine; Compound2, Octanal, 2-(2, 4-dinitrophenyl) hydrazone; Compound3, Deoxyshikonin; Compound4, Kavahin; Compound5, Dodecyl gallate*.

We also detected the combined effects of the five compounds and Erythromycin/ Tetracycline against *S. pneumoniae*. As shown in Table 6, Deoxyshikonin, Kavahin or Dodecyl gallate showed significant synergic antimicrobial activity with erythromycin against *S. pneumoniae*. Deoxyshikonin or Dodecyl gallate showed significant synergic antimicrobial activity with tetracycline against *S. pneumoniae*.

**Table 6:** *****In vitro*** inhibitory activity of five respective compounds in combination with erythromycin or tetracycline against ***S. pneumoniae*****.

**Antimicrobial agent**	**Erythromycin**		**Tetracycline**	
	**FICI**	**Outcome**	**FICI**	**Outcome**
5′-(Methylthio)-5′-deoxyadenosine	2		0.9	
Octanal, 2-(2, 4-dinitrophenyl) hydrazone	2		0.75	
Deoxyshikonin	0.37	Synergy	0.125	Synergy
Kavahin	0.5	Synergy	0.51	
Dodecylgallate	0.31	Synergy	0.18	Synergy

We selected deoxyshikonin and dodecyl gallate for further evaluations because of their considerable synergistic antibacterial activity with PEN. The efficacy of these compounds against two clinical *S. pneumoniae* strains with a high incidence in children was determined (Table 7). Deoxyshikonin and dodecyl gallate caused a two-log reduction of 19A bacterial numbers and a one-log reduction in 19F bacterial numbers at 24 h. The results showed that deoxyshikonin and dodecyl gallate eliminated the bacterial colonization in the nasopharynx and lung of mice at 24 and 72 h after infection. In the 19A challenge model, the group injected with dodecyl gallate plus PEN showed a 1-fold reduction in log CFU bacterial load in the nasopharynx within 48 h compared with penicillin/compound group. In the 19F challenge model, the joint groups (deoxyshikonin plus PEN and dodecyl gallate plus PEN) showed a 1-log CFU and 1.5-log CFU reduction in bacterial load in the nasopharynx within 48 and 72 h, respectively. These results are consistent with the *in vitro* results and suggest that the two compounds can enhance the sensitivity of bacteria to PEN and be used combination with other antibiotic to combat the antibiotic-resistant bacteria infection.

**Table 7:** *****In vivo*** inhibitory activity of two compounds alone and combinations against clinical ***S. pneumoniae*****.

**Strains**	**Antimicrobial agent**	**Dose (mg/kg/d)**	**Mean log CFU at site of infection^a^**					
			**NW (*P*-value)**			**Lung (*P*-value)**		
			**24 h**	**48 h**	**72 h**	**24 h**	**48 h**	**72 h**
19A	Control		7.29	5.97	6.01	4.85	6.12	6.29
	PEN	0.29	6.44	5.88	5.34	4.8	4.5	4.89
	Deoxyshikonin	0.45	6.08 (0.026)^b^	2.76 (0.005)^b^	3 (0.011)^b^	1	2.39 (0.0001)^b^	1.05 (0.04)^b^
	Dodecylgallate	0.57	5.91 (0.021)^b^	5.28	3.62 (0.033)^b^	1	3	2.82
	Deoxyshikonin+PEN		5.82	4.2	3.36	1.61	4.15	3.43
	Dodecylgallate+PEN		6.15	4.8 (0.04)^c^	3.35	1.61	2.25 (0.017)^c^	1.53
19F	Control		6.72	6.27	7.09	2.82	7.57	4.95
	PEN	0.16	6.21	2.1	6.12	1	2.58	3.22
	Deoxyshikonin	0.45	3.30	4.6 (0.0002)^b^	5.52 (<0.0001)^b^	1 (<0.0001)^b^	1 (0.001)^b^	2.5 (<0.0001)^b^
	Dodecylgallate	0.57	2.16	5.55	5.82 (<0.0001)^b^	3.9 (0.0001)^b^	3 (0.006)^b^	2.8 (<0.0001)^b^
	Deoxyshikonin+PEN		6.27	3.1 (0.049)^c^	5.34 (0.02)^c^	3.57	1	2.39
	Dodecylgallate+PEN		6.33	1.95	4.26 (0.004)^c^	7.5	3.45	2.51

a *The mice were intranasally infected clinical S. pneumoniae 19A (CMCC31689) and 19F (CMCC31693) (*1.5* × *10*^8^ CFU) in each group and received intraperitoneal injections of drugs three times a day for 3 days. The numbers of bacteria in nasopharynx and lung were determined at 24, 48, and 72 h*.

b *Differences between treated groups and controls were analyzed. P < 0.05 was considered significant*.

c *Differences between treated groups versus Penicillin. P < 0.05 was considered significant*.

### Effects of the five compounds on cell division phenotype

**Figure 4:**
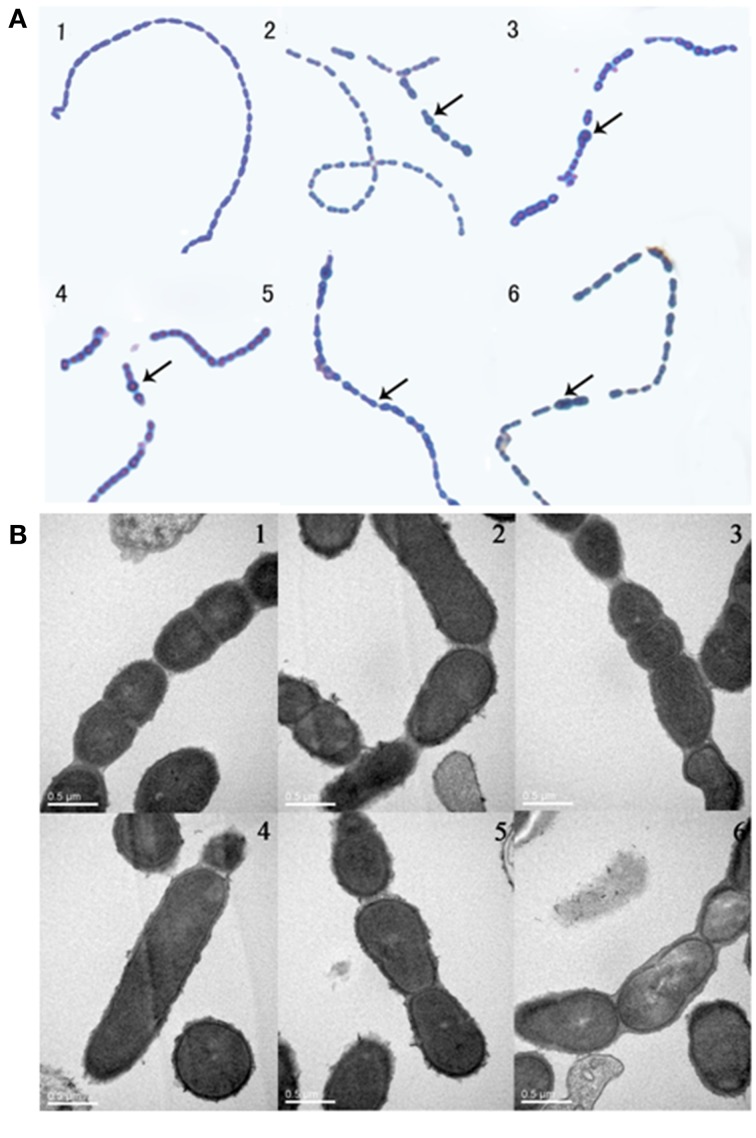
**The effect of the compounds on cell division**. Mid-exponential grown cells of D39 were diluted (1:1000) and incubated in the absence (1) or presence of compounds 1 (2), 2 (3), 3 (4), 4 (5), and 5 (6) for 5 h at 37°C. **(A)** Bacterial cells were observed by microscopy after staining with leather blue dye solution. **(B)** Bacterial cell were observed by transmission electron microscopy. Abnormal cells were indicated by black arrow.

**Figure 5:**
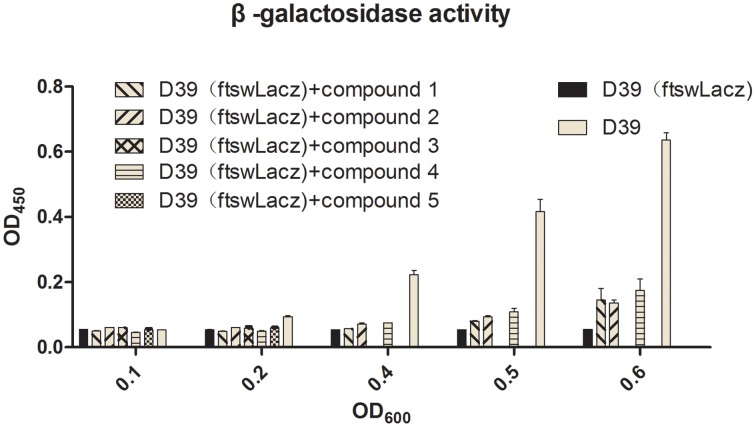
**The effects of the five compounds on the expression of FtsW**. LacZ were used to report the expression of FtsW. The β-galactosidase activities of the D39 (FtswLacZ) were determined following the treatment of the compounds. Wild-type D39 and D39 (FtswLacZ) were served as negative controls.

Barendt SM and Sham LT reported that VicK regulates the division of bacterial cells. In this study, bacteria were exposed to the agents at sub-MIC concentrations. Light microscopy and electron microscopy results showed that some bacteria became longer or thicker (Figures 4A5,A6,B2,B4), irregular or with abnormal divisions (Figures 4A2–A4,B3,B5,B6). Based on these findings, we detected the expression of FtsW which is an essential membrane protein and is involved in bacterial cell division (Dubrac et al., 2008). β-galactosidase activity was used to report the expression level of FtsW (Figure 5). In the control group, the expression of FtsW was increased as the optical density of the bacteria increased. When the strain was incubated with the five compounds, compared with that in the control group, the expression of FtsW was decreased regardless of the increase or decrease in growth density. The results suggested that the antibacterial activity of the five VicK inhibitors may partly attribute to the inhibition of FtsW.

### Inhibition of biofilm formation

**Figure 6:**
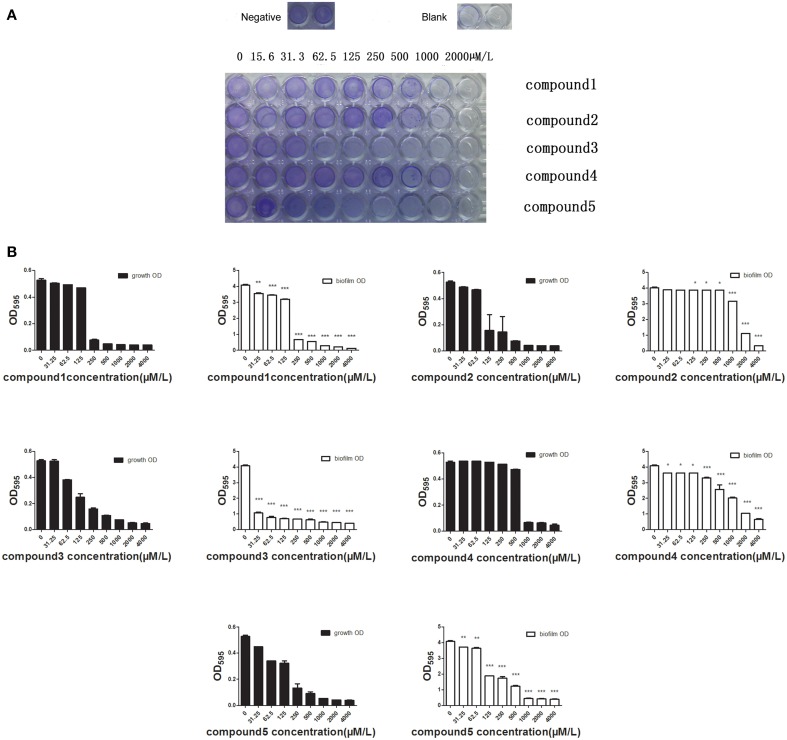
**The effect of the compounds to inhibit the formation of the bilofilm. (A)** Biofilms were visualized by crystal violet staining. The formation of biofilm can be seen in wells of bacteria treated with 1% DMSO (negative control) and the well without adding anything served as blank. The images are representative of results from three independent experiments. **(B)** The absorbance of the wells at 590 nm was detected using a spectrophotometer. The differences between the negative control (0 μM antimicrobial compounds) and antimicrobial-treated groups were statistically significant.

A recent study has reported that more than 60% of all bacterial infections in human are caused by microbial growth as biofilms, which show low susceptibility to antimicrobial agents (Moscoso et al., 2009). We determined the inhibitory activity of the five compounds against biofilms at different concentrations (15.6–2000 μM). Crystal violet staining revealed that all the studied compounds at MIC or sub-MIC levels inhibited the biofilm formation of *S. pneumoniae* NCTC7466 (Figure 6A). It's much more impressive for Deoxyshikonin to inhibit biofilm formation compared to other compounds. Crystal violet results were confirmed by determining the biofilm OD (Figure 6B). The results clearly showed that the compounds have the capacity to inhibit the development of biofilm formation.

## Discussion and conclusion

The emergence of *S. pneumoniae* strains resistant to carbapenem and vancomycin which are important agents for the treatment of gram-positive bacteria has become a global concern (Hanna-Wakim et al., 2012). Commonly, conventional antibiotics were designed based on their inhibitory effects against proteins with essential functions. In this study, the five compounds show an effective antimicrobial activity against the pneumococcus by targeting the upstream regulatory proteins of TCSs. The present work opens sights for the development of new therapeutic agents that may be effective against multidrug-resistant bacteria (Cai et al., 2002).

The active compounds of natural products are important sources of new drugs, and it's estimated that about half of the clinical drugs stemmed from natural products and their derivatives. TCM has a wide variety of active ingredients for discovering novel leading compounds. Several studies have reported the screening of antibacterial drugs from Traditional Chinese Medicines (Palacios-Espinosa et al., 2014). This is the first time to describe a specific target-based screening and identification of antibacterial compounds from TCM.

In the present study, we focus on the effects of the five TCM monomers on phosphatase activity of VicK, their antibacterial activity, their synergistic actions with penicillin. *In vitro* inhibition assays show that all of the five compounds can specifically inhibit the autophosphorylation of VicK protein (Figure 2). We have previously reported the ATP binding site of VicK consists of a hydrophobic inner cavity and a larger hydrophilic outer cavity (Li et al., 2009). It seems that all of the compounds share a common feature that they have a hydrophilic part containing several –OH and/or –NH moieties that are prone to form an intermolecular hydrogen bond; meanwhile, the hydrophobic part of these compounds is able to enter into the hydrophilic outer cavity of VicK. This may result in disorder of the VicK activity.

As expected, antibacterial results show that all of the five compounds have obvious inhibitory effects against *S. pneumoniae* D39 strain which is susceptible to all kinds of antibacterial agents (Figure 3). However, the emergency of drug-resistant pneumococcal strains has complicated the clinical treatment of pneumococcal infection. Therefore, we investigated the activities of the five compounds against penicillin- resistant *S*. *pneumoniae* (PRSP) strains *in vitro* (Table 2). The five compounds had antibacterial effects on all tested strains. Compared to the other three compounds, deoxyshikonin, and dodecyl gallate were more effective against the tested strains, including the important pathogen PRSP. These data indicate that the five compounds could be an effective alternative to treat drug-resistant pneumococcal strains. In addition, we found that five compounds were also effective against *S. mutans, S. pyogenes, S. mitis*, and *S. pseudopneumoniae* (Table 3). The homology of VicK protein in these bacteria with *S. pneumoniae* was up to 81–95% (http://blast.ncbi.nlm.nih.gov/Blast.cgi), which could be used to explain why these compounds have a broad spectrum activity in streptococci.

Although sequence alignment with BlastP demonstrated that *S. aureus* VicK shares 68% amino acid similarity with its counterparts in *S. pneumoniae* (http://blast.ncbi.nlm.nih.gov/Blast.cgi), not all of the five compounds but deoxyshikonin and dodecyl gallate inhibited the growth of *S. aureus* strains (Table 2). We hypothesized that the center conformation of VicK ATPase in *S. aureus* is different from that in streptococci and hence led to the discrepancy in inhibition between *S. aureus* and streptococci. However, it's also possible that the target of deoxyshikonin or dodecyl gallate may be not limited to VicK in *S. aureus*. Further studies are required to distinguish these possibilities.

VicR/K can affect bacterial cell division by regulating the expression of DacA, Pmp23, DacB, and FtsX in *S. pneumoniae* (Dubrac et al., 2008; Sham et al., 2011). In the present study, we observed that bacteria were defective in cell division following treatment with the compounds (Figure 4). FtsW is also an essential membrane protein involved in bacterial cell division, and we showed that the expression of FtsW was impaired following the treatment with the five compounds (Figure 5). The data are in accordance with a regulation of FtsW by VicK. These results indicate that the five VicK inhibitors could impair cell division of the bacteria via FtsW.

Bacteria in biofilms are 100–1000 times more resistant to antibiotics than planktonic cells (Kubo et al., 2003; Passerini de Rossi et al., 2009). VicR/K can also regulate the bacterial biofilms formation (Gupta and Kohli, 2003; Qin et al., 2007; Mun et al., 2013). As expected, five compounds also displayed strong bactericidal activities toward cells in biofilms. Particularly, deoxyshikonin and dodecyl gallate could inhibit biofilm formation at sub-MIC. A previous study reported that *S. pneumoniae* TCS11 and TCS12 (comDE) were implicated in the formation of biofilm (Cockeran et al., 2014; Galante et al., 2015). HK11 and comD only show a discernable similarity with VicK protein, and the similarity is less than 30% even in regard to the ATPase domain. Thus, these compounds may have, if any, a very limited effect on inhibition of the HK of the two TCS, and biofilm inhibition by these compounds may be primarily attributed to their inhibitory effect on VicK protein.

Several studies have proposed that the combination of natural compounds with antibiotics to treat infections caused by bacterial species and natural plant products may potentiate the activity of antibiotics (Garo et al., 2007). Their combinations may enhance antibacterial efficacy and reduce the emergence of antibiotics-resistant strains. Given that the different antibacterial mechanism between the VicK inhibitors and the conventional antibiotics, such as PEN, erythromycin and tetracycline, they are used in combination to explore whether they have synergistic or additive effects against pneumococcal infections. Our results showed that deoxyshikonin or dodecyl gallate in combination with PEN had synergistic activities against all the multidrug-resistant *S. pneumoniae* strains *in vitro* (Table 5) and *in vivo* (Table 7). However, the synergistic effect of the combinations of the other three compounds with these antibiotics was not obvious. Nevertheless, their MICs could be reduced, suggesting the three compounds also have enhancement effects on antibiotics. Interestingly, deoxyshikonin, kavahin, or dodecyl gallate showed significant synergic antimicrobial activity with erythromycin against *S. pneumoniae* (Table 6). *S. pneumoniae* is one of the most important human pathogens in children. Erythromycin drugs are the first choice for children allergic to penicillin. However, the erythromycin resistance rate in *S. pneumoniae* isolates is significantly high (Ma et al., 2013), thus, the three compounds may have potential prospect to treat erythromycin resistant bacteria.

These compounds significantly prolong the survival times of mice infected with lethal dose of pneumococci in animal models (Table 4). Dodecyl gallate was also effective against intranasal infection with *S*. *pneumoniae* 19F strain, except that dodecyl gallate-treated mice exhibited higher bacterial load in the lungs than control mice at 24 h. It seems that *in vivo* antibacterial activities were not always correlated with their MIC values, and this may be explained by the fact that the antibacterial effects of drugs may be affected by absorption, distribution metabolism and metabolism *in vivo* (Neve et al., 2013; Cõrte-Real et al., 2014);. Therefore, to obtain more effective inhibitors, structural modification of these compounds may be required in the future work.

Protein phosphorylation signal transduction system is widespread in bacteria, such as Ser/Thr/Tyr phosphorylation. Bacterial tyrosine kinases would be a promising target for the screening of antibacterial drugs since they do not share any similarity with those of eukaryote. A recent report shows that phosphorylation of proteins in carbapenem resistant strains of *A. baumannii* is significantly higher than that in carbapenem sensitive strains (Tiwari and Tiwari, 2015). Thus, targeting the kinase may provide a potentially useful way to discover antimicrobial compounds that are effective against antimicrobial agents-resistant bacteria. Together with our data support the concept that it would be useful to screen the potential antibacterial drugs by targeting kinases.

Taken together, the present study provides preclinical evidence of the efficacies of the five lead compounds against the pneumococcus and other gram-positive bacteria. These novel antibacterial agents may serve as an alternative strategy for the treatment of bacterial infections.

### Conflict of interest statement

The authors declare that the research was conducted in the absence of any commercial or financial relationships that could be construed as a potential conflict of interest.

## Abbreviations

TCM: Traditional Chinese Medicine
PEN: penicillin
MIC: minimal inhibitory concentration
TCS: Two-component systems
PRSP: penicillin-resistant *S. pneumoniae;* MRSA, methicillin-resistant *Staphylococcus aureus*.
